# MYCN and the epigenome

**DOI:** 10.3389/fonc.2013.00001

**Published:** 2013-01-25

**Authors:** Stanley He, Zhihui Liu, Doo-Yi Oh, Carol J. Thiele

**Affiliations:** Cell and Molecular Biology Section, Pediatric Oncology Branch, Center for Cancer Research, National Cancer InstituteBethesda, MD, USA

**Keywords:** MYCN, EZH2, epigenetics, nucleosome, RNA polymerase II, HDAC, HAT, transcriptional activation

## Abstract

It is well known that Neuroblastoma (NB) patients whose tumors have an undifferentiated histology and a transcriptome enriched in cell cycle genes have a worse prognosis. This contrasts with the good prognoses of patients whose tumors have histologic evidence of differentiation and a transcriptome enriched in differentiation genes. Tumor cell lines from poor prognosis, high-risk patients contain a number of genetic alterations, including amplification of MYCN, 1pLOH, and unbalanced 11q or gains of Chr 17 and 7, and exhibit uncontrolled growth and an undifferentiated phenotype in *in vitro* culture. Yet treatment of such NB cell lines with retinoic acid results in growth control and induction of differentiation. This indicates that the signaling pathways that regulate cell growth and differentiation are not functionally lost but dysregulated. Agents such as retinoic acid normalize the signaling pathways and impose growth control and induction of differentiation. Recent studies in embryonic stem cells indicate that polycomb repressor complex proteins (PRC1 and PRC2) play a major role in regulating stem cell lineage specification and coordinating the shift from a transcriptome that supports self-renewal or growth to one that specifies lineage and controls growth. We have shown that in NB, the PRC2 complex is elevated in undifferentiated NB tumors and functions to suppress a number of tumor suppressor genes. This study will review the role of MYC genes in regulating the epigenome in normal development and explore how this role may be altered during tumorigenesis.

## MYCN AND NEUROBLASTOMA

Ever since MYCN was discovered to be the commonly amplified sequence in neuroblastoma (NB) tumors some 30 years ago, the mechanisms by which it contributes to NB tumorigenesis and strategies to target it have been intensively investigated (Schwab et al., 1983). The finding that MYCN amplification in NB tumors identified patients with the worst prognoses led to the integration of MYCN copy status into risk stratification protocols for treatment (Goto et al., 2001). MYCN was the first oncogene integrated into clinical practice and is still used to stratify patients for therapy. One of the early attempts to target MYCN evolved from the finding that retinoids inhibited MYCN expression as part of the widespread transcriptional changes retinoids induced in NB cells which resulted in tumor cell growth arrest and differentiation (Thiele et al., 1985). Later retinoids were integrated into the consolidation phase of high-risk therapeutic protocols and found to increase patient overall survival (Park et al., 2009). However, the molecular mechanisms by which MYCN stimulates tumorigenesis have remained a mystery.

Functionally MYC family genes (MYC, MYCN, and MYCL) have been implicated in nearly every biologic process evaluated. Evidence indicates that MYC and MYCN are functionally interchangeable with MYCN having a more restricted spatial and temporal role during development (Malynn et al., 2000). In this review, it is assumed that what is known for the molecular mechanisms of MYC action can be extrapolated to MYCN, unless otherwise noted. This review will focus on MYC interaction with the epigenome especially with regards to regulation of gene transcription and translation.

## MYC, CHROMATIN STRUCTURE, AND GENE TRANSCRIPTION

**FIGURE 1:**
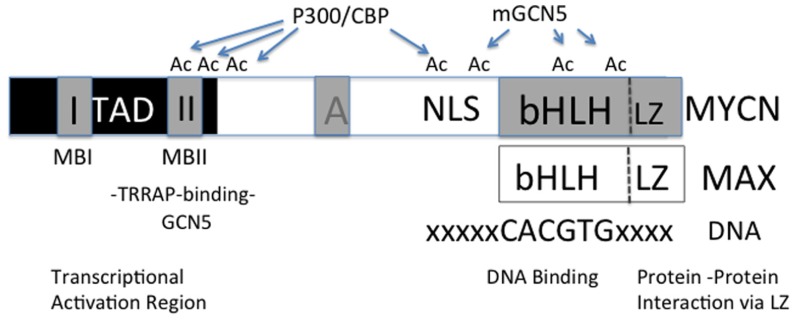
**Diagram of MYC functional regions**. In the region of the transcriptional activation domain (TAD) the MYC Box II (MBII) binds TRRAP containing complexes of proteins that may also contain GCN5. It is also felt that p-TEFb, Tip60, and a number of other transcriptional regulatory factors can bind to this region. Determinants of which factors bind remain to be resolved. The bHLH region binds E-box containing regions of DNA. MYC dimerizes with other bHLH containing transcription factors via a carboxy-terminal leucine zipper (Zip) region. While MYC recruits HATs such as GCN5 and CBP/p300 which are known to acetylate lysine residue on histone tails, these HATs are also known to acetylate (Ac) MYC which affects its stability by blocking or competing with ubiquitin ligases that target MYC for degradation.

MYC is a member of a basic helix-loop-helix transcription-leucine zipper (bHLH-Zip) factor family whose members dimerize with MAX and bind to specific DNA sequences called E-boxes (**Figure 1**). Based on studies using naked DNA, two prevailing views emerged as to how MYC affects transcription: (1) a direct mechanism in which MYC/MAX dimers upon binding to E-boxes recruit higher order chromatin complexes that activate or repress transcription or (2) an indirect mechanism in which MYC/MAX dimers compete with other bHLH-Zip transcription factors for E-box binding to alter target gene activity. In searching for a common MYC target signature investigators have proposed numerous target signatures in different cellular contexts, but none have proven true in all cellular contexts (Cotterman et al., 2008; Soufi et al., 2012).

Recent studies assessing global transcription regulated by MYC in normal cells as well as in tumor cells reveal that MYC acts as a direct amplifier of transcriptionally active genes and does not directly induce *de novo* gene transcription or directly silence expressed genes (Lin et al., 2012; Nie et al., 2012; Soufi et al., 2012). MYC function as a transcriptional amplifier is supported by the lack of a classical transcriptional gene signature in numerous contexts, although, the precise molecular mechanisms that mediate transcriptional amplification remains to be elucidated (Knoepfler et al., 2006; Nie et al., 2012). Given, the lack of a classical transcriptional signature and the apparent dependence of MYC on chromatin context, studies have suggested MYC elicits its function epigenetically (Guccione et al., 2006; Soufi et al., 2012).

Within the last decade there has been a dramatic shift in our understanding of mechanisms of transcriptional regulation. This, coupled with the ability to query the entire transcriptome has given us a broader understanding of how MYC affects transcription. In order to appreciate MYC’s role in epigenetic regulation we must first step back and look at the problem of higher order chromatin structure and regulation of gene transcription.

**FIGURE 2:**
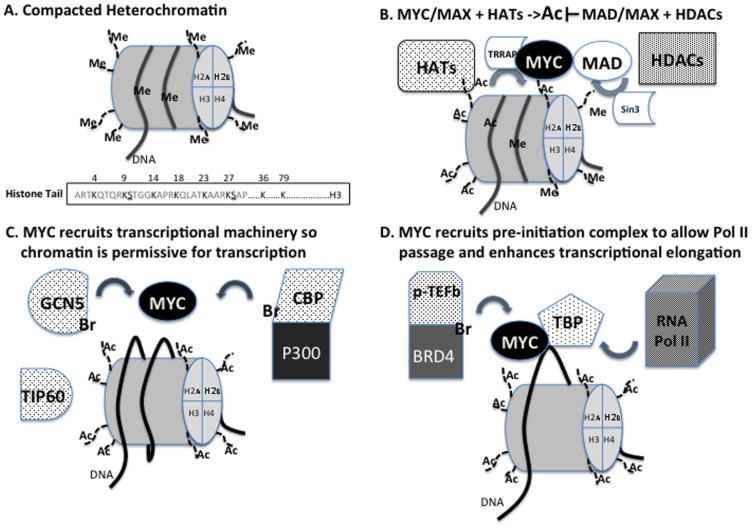
**(A)** Compacted heterochromatin is characterized by specific methylation of H3K9 and H3K27 is often correlated with decreased transcription. The EZH2 component of PRC2 contains the H3K27methylase while EHMT2 or G9a contains the H3K9 methylase. The methylation status of these amino acids is regulated by demethylases such as LSD1, JMJD2, and UTX. The subsequent acetylation (Ac) of H3K9 by GCN5 and H3K27 by CBP/p300 are associated with relief of gene suppression. **(B)** MYC recruits TRRAP to target loci to mediate MYC binding to histone acetyltransferases (HATs). Recruited HATs acetylate histone components to promote transcription. Conversely, MAD recruits Sin3 to target loci to mediate MAD binding to histone deacetylases (HDACs). Histone deacetylation allows for methylation events leading to transcriptional silencing. **(C)** Via TRRAP, MYC is able to recruit HATs GCN5, TIP60, and CBP/p300 to target loci to relax the nucleosomal barrier making DNA more accessible to transcriptional machinery and making the nucleosome more permissive for transcription. **(D)** MYC can also bind to TBP suggesting it can recruit elements of the transcriptional pre-initiation complex to initiate transcription. To maintain transcription MYC recruits p-TEFb and BRD4 to catalyze promoter pause release and transcriptional elongation. BRD4, as well as GCN5 and CBP, have bromodomains (Br) that recognize acetyl-lysines which has proven necessary for proper function.

The necessity to compact some 2 m worth of DNA into the 10–20 μm nucleus of a cell has resulted in the evolution of a dynamic packaging system in eukaryotes that allows for the regulated sequestration or exposure of stretches of DNA. DNA (about 146 bp) is wrapped twice around an octamer of histone proteins (H3, H4, H2A, and H2B) and stabilized by histone H1, forming the nucleosome, the basic unit of chromatin (**Figure 2A**). Nucleosomes are then joined by a stretch of linker DNA (20 bp). When DNA is tightly complexed to histones, transcription is silenced. Activation of gene transcription requires loosening of DNA–histone interactions to enable access by transcription factors, the melting of the DNA that enables access of the basal transcription machinery including RNA polymerase II (Pol II) and the sliding of the nucleosomes to enable transcriptional elongation. The accessibility of DNA to DNA binding transcription factors is dynamically regulated by post-translational modifications to histone “tails” such as methylation (methyltransferases and demethylases), acetylation (acetyltransferases and deacetylases), phosphorylation (kinases and phosphatases), ADP-ribosylation, and ubiquitination (**Figure 2A**).

The diversity of histone post-translational modifications led to the proposal that they represented a “histone code” that was written and erased by various enzymes or protein complexes and functioned to regulate the accessibility of DNA for DNA replication or gene transcription (Strahl and Allis, 2000). In this model, the combinatorial power of the various modifications would enable the integration of stimuli from numerous different environmental signaling pathways and serve as a final readout directing gene activation or suppression. The model also seems to reconcile apparent situational discrepancies in which post-translation modifications such as acetylation may be associated with gene activation or suppression depending on cell lines or context. While global histone acetylation may correspond to active transcription, downstream effectors activated may instigate suppression at certain loci. Not only do the modifications affect the charge of the histones and their interaction with DNA but they also can be read or serve as docking sites for other proteins. For example, acetylated histones serve as docking sites for bromodomain (Br) containing proteins (Dhalluin et al., 1999; Dey et al., 2003). Thus, the histone code imparts a tertiary level of genomic control beyond the DNA sequence and corresponding transcription factors.

## MYC AND CHROMATIN REMODELING

### HISTONE ACETYLATION

Nucleosomal elements of the epigenome not only control DNA accessibility, but also function as a physical barrier to transcription that transcription factors and chromatin remodeling complexes must overcome to initiate and maintain transcription (Zaret and Carroll, 2011; Bintu et al., 2012). Emerging evidence suggest transcription factors and chromatin remodeling complexes coordinate to access target DNA loci and conversely silence loci when the gene product is no longer desired (Cirillo et al., 2002; Filion et al., 2010; Zaret and Carroll, 2011; Soufi et al., 2012). While epigenetic functions of MYC are still being elucidated, recent studies demonstrate MYC may direct epigenetic changes at target loci by relaxing the nucleosomal barrier making it more permissive for subsequent transcription.

When MYC function was first being described over 20 years ago, it was noted that MYC preferentially binds to “open” nucleosomes and DNase sensitive loci that we now know characterize euchromatin (Klempnauer, 1989; Telford and Stewart, 1990). Euchromatin was known to correlate with active transcription, but how MYC modulated this effect was unclear. As studies emerged explicating histone modifications, it was shown that MYC recruits macromolecular complexes containing chromatin remodelers to its target loci (McMahon et al., 1998, 2000; Cheng et al., 1999; Wood et al., 2000; Frank et al., 2003; Vervoorts et al., 2003). Evidence suggested MYC elicited its oncogenic effects by binding to cofactors via a highly conserved amino acid domain in its N-terminus transactivation domain called MYC Box II (MBII; **Figure 1**; Stone et al., 1987). Seminal work discovered that a novel protein, transformation/transcription domain-associated protein (TRRAP), binds to MYC at MBII and deletion of MBII or inhibition of TRRAP abrogated MYC induced oncogenic transformation (McMahon et al., 1998). These results implicated TRRAP as an essential cofactor for MYC function, but TRRAP itself had no histone remodeling function (**Figure 2B**).

Further work revealed that, via TRRAP, MYC was able to recruit histone acetyltransferases (HATs), GCN5 and Tip60 to its targets. GCN5 and Tip60 were first identified in *Saccharomyces cerevisiae* as members of the SAGA and NuA4 HAT complexes, respectively, and have been shown to have similar function in mammals (McMahon et al., 2000; Frank et al., 2003; **Figure 2C**). Concordantly, in embryonic stem cells (ESCs) MYC has been shown to elicit its transcriptional induction through the mammalian NuA4 complex and in neural stem cells MYCN and GCN5 have similar transcriptional signatures (Kim et al., 2010; Martinez-Cerdeno et al., 2012). Both GCN5 and Tip60 have HAT activity, but differ in their histone substrate specificity. GCN5 preferentially acetylates H3, H2B, while Tip60 preferentially acetylates H4 and H2A (Allard et al., 1999; McMahon et al., 2000). In addition, Myc has also been shown to bind to TATA-binding protein (TBP), a member of the transcriptional pre-initiation complex, and cAMP-response-element-binding protein (CBP), one half of the CBP/p300 coactivator complex, which has HAT activity and scaffolding functions (McEwan et al., 1996; Ogryzko et al., 1996; Chan and La Thangue, 2001; Vervoorts et al., 2003; **Figure 2C**). Although TBP has no HAT activity, its binding to MYC suggests MYC is able to recruit Pol II machinery to activate transcription (**Figure 2D**). Therefore, through its binding partners MYC is able to regulate the chromatin landscape to exert its function.

The functional impact of acetylated histones is a general reduction to the nucleosomal barrier, promoting cofactor binding, and increased Pol II passage, thereby increasing transcriptional activity (Knoepfler et al., 2006; Bintu et al., 2012; **Figure 2D**). GCN5 and Tip60 function to promote transcription by their acetyltransferase activity, but it is not limited to histones. It has also been demonstrated that MYC is a substrate of acetyltransferase activity. GCN5, Tip60, and CBP/p300 all acetylate MYC (**Figure 1**; Patel et al., 2004). Acetylation of MYC stabilizes the protein and prevents ubiquitin-mediated degradation thereby increasing MYC transcriptional latency (**Figure 1**; Vervoorts et al., 2003; Patel et al., 2004). These results suggest that not only are the HATs MYC recruits to its target loci essential transcriptional cofactors, but also essential to maintain MYC function in a pseudo-feed-forward mechanism.

Convincingly, MYC has been shown to recruit chromatin modifiers to elicit its transcriptional amplifier function. Less well resolved is how amplified MYC transcripts further act as downstream effectors. In neural progenitors, MYC has been shown to stimulate the transcription of GCN5, which endows MYC with a feed-forward loop stimulating increased transcriptional activity and its own stability (Knoepfler et al., 2006). It is of interest that in neural progenitors both GCN5 and MYCN conditional knockouts have impaired precursor proliferation *in vitro* and are marked by microcephaly. Moreover, the transcriptomes of GCN5 and MYCN conditional knockouts exhibit overlap in a significant number of repressed and activated genes that commonly are involved in signaling and neural differentiation (Martinez-Cerdeno et al., 2012).

### ATP-DEPENDENT CHROMATIN REMODELING

In addition to HAT recruitment via MBII binding to TRRAP, MYC was also shown bind to INI1, a member of the SWI/SNF ATP-dependent chromatin remodeling complex. This interaction is mediated via MYC’s bHLH and Zip domains and INI1’s Rpt1 domain (**Figure 1**). As MYC’s bHLH domain is required for its transactivation function and binding to INI1, INI1 appears indispensable for proper MYC function. Furthermore, intact SWI/SNF complexes are also needed for MYC function. BRG1 is a functional component of the SWI/SNF complex and its ATPase-defective mutant abrogates histone remodeling activity and blocks transcription of MYC targets (Cheng et al., 1999). It has also been shown BRG1 can antagonize MYC transcriptional activation in cancer models (Romero et al., 2012). While the results in tumor models suggest BRG1 is necessary for maintenance of proper gene expression and response to environmental stimuli, the abrogation of BRG1 most likely blocks nucleosomal responses initiated not only by MYC, but by other chromatin remodeling complexes as well.

## MYC ENHANCES TRANSCRIPTIONAL ACTIVATION

By the recruitment of HATs and the SWI/SNF complex, MYC can remodel the nucleosomal topography to promote transcription. But the question remains as to whether MYC can initiate transcription. Results have shown that MYC cannot initiate *de novo* transcription, but is necessary to maintain a nucleosomal landscape permissive for transcription (Knoepfler et al., 2006; Soufi et al., 2012). Extensive chromosome immunoprecipitation and sequencing (ChIP-Seq) analysis has revealed MYC E-box binding is dependent on chromatin context. Without specific euchromatic islands characterized by methylated H3K4 and H3K79 and acetylated H3, MYC is unable to access E-boxes on its target loci (Guccione et al., 2006). Intriguingly, MYC also binds to euchromatic islands not on E-boxes, which may also explain MYC E-box binding degeneracy.

In NB, a genome-wide assessment of MYCN using the tetracycline (TET) suppressible MYCN SHEP NB model system and ChIP hybridizations to gene promoters (ChIP-Chip) revealed that MYCN binding associates with euchromatic histone marks H3K4me and H3K9Ac (Cotterman et al., 2008). The correlation of MYCN binding and microarray analyses revealed that when MYCN was induced, MYCN was found bound to genes that were already transcriptionally active. MYCN was not associated with *de novo* gene transcription. This is consistent with the recent models of MYC binding at or near transcription start sites (TSS) and functioning to amplify transcription of actively transcribed genes (Lin et al., 2012; Nie et al., 2012; Soufi et al., 2012). An interesting aspect of the Cotterman’s study was the finding that upon loss of MYCN expression there was a global loss of the euchromatic marks even at sites that were not bound by MYCN (Cotterman et al., 2008). This implies that MCYN plays a role in regulating euchromatic regions in a manner that is independent of its role as a classic transcription factor. One caveat raised in this study was whether the assays were sufficiently sensitive to detect low affinity MYCN binding sites. The utilization of more sensitive and quantitative technologies such as MYCN ChIP-seq should clarify this in the future.

MYC dependence on chromatin context is further supported by studies revealing the temporal basis for chromatin remodeling. During cellular reprogramming ectopic expression of transcription factors can induce global chromatin changes as a cell reverts from a differentiated state to a pluripotent state. Temporal analysis of reprogramming revealed that among the reprogramming factors Oct4, Sox2, Klf4, and MYC (OSKM), only OSK can bind to distal enhancer regions at an early time during reprogramming. Subsequently MYC is recruited to the TSS to stabilize chromatin binding and promote transcription. Furthermore, OSK proteins are all able to bind to one side of the DNA helix, suggesting they are able to bind to heterochromatic regions. MYC does not bind to heterochromatic regions. MYC is predominantly found on euchromatic regions indicating it requires a prior transcriptional stimuli or *de novo* factors to bind to DNA to initiate its transcriptional function (Soufi et al., 2012).

Since MYC cannot initiate *de novo* transcription, the question arises as to how MYC can promote the induction of transcription initiated by reprogramming factors. Regulation of gene transcription in complex chromatin models is still evolving and occurs in at least five steps: (1) initiation, (2) promoter pausing, (3) mRNA capping, (4) promoter escape, and (5) transcript elongation. A clue to MYC function during initiation lies with MYC binding to TBP (**Figure 2D**; McEwan et al., 1996). MYC is a direct TBP binding partner and may be able to couple the pre-initiation complex to its target promoters. Moreover, MYC has been implicated at mRNA capping, promoter escape, and elongation.

Until recently, the predominant or rate limiting mechanism regulating initiation of transcription on gene promoters was thought to be the binding or loading of Pol II onto target DNA sites, termed initiation. Pol II bound at promoters is marked by phosphorylation by TFIIH on serine 5 of its carboxy-terminal domain (CTD), p-Ser5-Pol II. Genome-wide analyses of Pol II binding demonstrated that p-Ser5-Pol II commonly resides at promoters or is paused 20–40 bases downstream of the promoters of many non-expressed genes. The capping machinery is recruited to the CTD of Pol II and results in the addition of a methylated guanine at the 5′-triphosphate of the nascent mRNA strand. MYC also binds to TFIIH and the increased p-Ser5-Pol II increases capping associated methylation. Capping is required for the recruitment of the translation machinery and mRNA binding to ribosomes. The acetylated lysine binding bromodomain containing protein BRD4, recruits P-TEFb complex which phosphorylates serine 2 on the CTD region of Pol II (p-Ser2-Pol II) causing elongation of mRNA transcripts and promoter escape (**Figure 2D**; Rahl et al., 2010). MYC also binds to P-TEFb enhancing P-TEFb kinase activity. While MYC effects on transcriptional elongation are dependent on its DNA binding activity, evidence indicates that MYC effects on capping are independent of its DNA binding activity (Cole and Cowling, 2008; Cowling and Cole, 2010).

**FIGURE 3:**
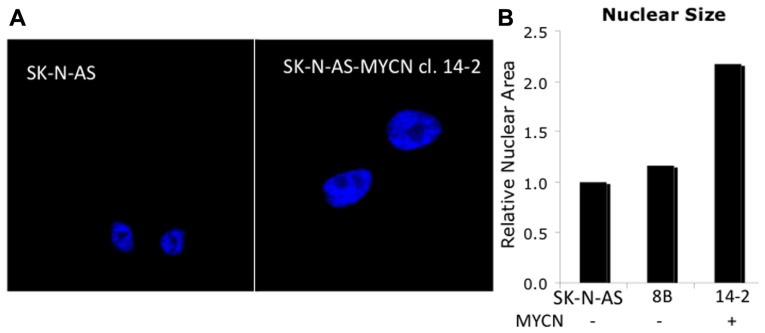
**MYCN influences global chromatin structure**. **(A)** The SK-N-AS cell line contains a single copy of MYCN and does not express significant quantities. Upon transfection of SK-N-AS with MYCN (14-2, Vector control: 8B), one can see enlarged nuclei in high MYCN expressing cells (nuclei are stained with DNA binding dye DAPI). **(B)** Measurements indicate a twofold increase in nuclear size.

By studying reciprocal events in differentiation and reprogramming the progression from transcriptional initiation to elongation can be extrapolated and pathologic consequences can be evaluated. During reprograming OSK function much like pioneering factors during embryonic development and differentiation. Pioneer factors initially bind to heterochromatic regions and then recruit cofactors and other transcription factors to initiate transcription (Cirillo et al., 2002; Zaret and Carroll, 2011). MYC would then be recruited to promoter regions to reinforce the permissive chromatin state. In neural progenitors loss of MYC results in increased H3K9me2, a mark of repressive chromatin, decreased acetylated H3 and H4, marks of active chromatin, and overall chromatin condensation characterized by increased heterochromatin. The changes in histone architecture are also noted to be cell cycle and differentiation independent, but higher MYC expression did correlate with increased nuclear size (Knoepfler et al., 2006). This change in nuclear size can be seen in **Figure 3A**, in which MYCN transfection into the NB cell line SK-N-AS (clone 14.2) results in cells with a relative nuclear size twice that of the parental or control-transfected (8B) cell lines which contain a single copy of MYCN and do not express MYCN mRNA (**Figure 3B**). Decreased euchromatic regions have also been identified in tumor models when MYC is knocked down (Wu et al., 2007). The results suggest MYC is essential in euchromatin maintenance after its initial recruitment. In this context, the importance of MYC in neural progenitors may derive its importance from stimulating transcription of nascent factors necessary for neural proliferation before being down-regulated to initiate terminal differentiation.

### MYC AND TRANSCRIPTIONAL REPRESSION

As a transcriptional amplifier, transcripts targeted by MYC would be expected to be up-regulated, but there remains a sizeable cohort of down-regulated MYC targets. MYC does not bind to corepressor complexes, so it is reasonable to surmise the down-regulated genes are secondary or indirect effects of MYC function. A recent study by Valentijn et al. (2012) identified a MYCN gene signature set in NB cell lines of 157 genes in which a subset was transcriptionally repressed genes (Valentijn et al., 2012). ChIP analyses on promoter arrays indicated that MYCN exhibited relatively weaker binding to the TSS of repressed genes compared to binding at the TSS of up-regulated genes. This is consistent with the recent MYC ChIP-seq studies that did not find MYC binding to TSS of genes repressed by MYC expression (Lin et al., 2012; Nie et al., 2012).

Early models of transcriptional suppression associated with MYC and MYCN involve an indirect mechanism in which MYC/MAX dimers compete with other bHLH-Zip transcription factors for E-box binding to alter target gene activity. For example the MYC antagonist MAD also has chromatin modifying abilities that may explain how it can modulate MYC function. MAD is able to recruit histone deacetylases (HDACs), via SIN3 a transcriptional corepressor, to target loci to induce transcriptional silencing (Laherty et al., 1997; Ayer, 1999; Knoepfler and Eisenman, 1999). Thus the model arises where MYC/MAX recruit HATs to induce chromatin acetylation making it more permissive for transcription and MAD/MAX recruit HDACs to deacetylate chromatin thereby silencing transcription (**Figure 2B**). In NB, the combined treatment of RA and Interferon-gamma (INF-γ) has been shown to dramatically decrease cell growth and induce differentiation (Lucarelli et al., 1995). It was recently shown that the transcriptional repression of two genes associated with the growth of NB cells, ornithine decarboxylase (ODC) and human telomerase reverse transcriptase (hTERT) was associated with a shift in promoter occupancy of MYCN/MAX binding at steady-state to MAD/MAX binding after RA and INF-γ treatment. This results in a decrease in H4 acetylation at these promoters and decreased expression of ODC and hTERT mRNA (Cetinkaya et al., 2007). The precise levels of activation and silencing probably exist in stoichiometric balance and are dependent on environmental and cellular mechanisms regulating MYC and MAD expression.

Another model of MYCN-mediated transcriptional repression invokes the association of MYC binding to the MIZ and SP1 transcriptional activators resulting in the recruitment of HDACs. This has been demonstrated for two genes associated with good prognosis and differentiation in NB, NTRK1(TrkA), and p75(NGFR). Upon induction of MYCN, there is increased HDAC1 and decreased acetylated H3 binding over the TSS of these promoters. The functional presence of MYCN was found to attenuate their promoter activity (Iraci et al., 2011). One can see how the increased levels of MYCN that accompanies gene amplification would disrupt normal cellular transcription factor stoichiometry leading to aberrant gene regulation.

Most recently MYC has been found to transcriptionally amplify epigenetic modifiers with transcriptional repressive activities. For example, in ESCs MYC stimulates all components of the polycomb repressive complex 2 (PRC2), including embryonic ectoderm development (Eed), suppressor of zeste 12 (Suz12), and histone methyltransferase enhancer of zeste homolog 2 (EZH2), in ESCs (Zhang et al., 2005; Neri et al., 2012). PRC2 functions to epigenetically silence gene expression, but at a different histone residue. PRC2 catalyzes trimethylation of H3K27 (H3K27me3). It is believed that the persistent H3K27me3 at particular gene loci results in the recruitment of the PRC1 complex to induce chromatin compaction (reviewed in Bernstein et al., 2007; Simon and Kingston, 2009). In NB relatively high levels of EZH2 are associated with undifferentiated NB tumors (Wang et al., 2012). The dysregulation of EZH2 may be due to a number of factors including MYCN, increased chromosome 7 copy number or loss of a regulator miR-101, which resides on 1p36. Functionally EZH2 was reported to suppress a number of genes with tumor suppressor activity in NB including the MYCN-regulated genes CLU and p75(NGFR). Enhanced EZH2 and it target H3K27me3 binding were detected at potential MYCN binding sites at steady-state conditions but the addition of an HDAC inhibitor was associated with decreased EZH2 and H3K27me3 binding and increased CLU and p75 expression (Wang et al., 2012). It is not known whether PRC2 complex components directly bind to MYC/MYCN but the enhanced binding of EZH2 and H3K27me3 covered an E-box in the CLU promoter and was over the TSS area identified to bind MYCN in the p75(NGFR) promoter (Iraci et al., 2011).

Furthermore, in NB cell lines and tumor samples, MYCN stimulates B cell-specific Moloney murine leukemia virus integration site (BMI1) expression. BMI1 is a member of the PRC1 complex whose targets are often hypermethylated suggesting epigenetic silencing (Ochiai et al., 2010). As part of a suppressive complex, BMI1 has been shown to repress tumor suppressor genes and catalyze events leading to NB tumorigenesis. BMI1 is highly expressed in NB cell lines and NB tumor samples and was shown to have anti-apoptotic effects via stimulation of RING1A/B ubiquitin-mediated degradation of P53 (Cui et al., 2007; Calao et al., 2012). The pro-survival effects of BMI1 suggest MYCN induction leads to greater chances for tumor initiating events. Beyond BMI1’s transcriptional targets in NB, PRC1 has also been shown to inhibit the transcriptional pre-initiation complex thereby inhibiting Pol II-mediated transcription (Lehmann et al., 2012). The summation of the events suggests BMI1/PRC1 suppress active transcription and catalyze epigenetic silencing, which may lead to NB tumorigenesis.

Given MYCN’s role in stimulating expansion of neural progenitors, it is not unexpected that differentiation genes like NTRK1 or p75(NGFR) are repressed by MYCN. A global survey by ChIP-Chip of ESCs for MYC and Miz-1 binding sites indicated that almost 30% of Miz-1-regulated genes could be repressed by MYC. These included homeobox genes, developmental proteins and genes involved in regulation of apoptosis. In NB tumor samples, within the MYCN gene signature set, 30% of the down-regulated genes in the set are neuronal tissue-specific genes while only 2% of such genes are up-regulated (Valentijn et al., 2012). This is consistent with one of the first studies on MYCN regulation in NB cells which indicated that retinoid mediated down-regulation of MYCN occurred prior to evidence of differentiation (Thiele et al., 1985).

## TARGETING MYC

Since MYC has been implicated in oncogenic transformation, researchers and clinicians have sought to target MYC and its oncogenic functions. A common mode of function of therapeutic interest has been MYC’s chromatin remodeling and transcriptional activities. It was rationalized that since MYC binds and recruits coactivators containing the bromodomain and extraterminal (BET) members of human bromodomain proteins (BRD2, BRD3, and BRD4), which associate with acetylated histones, it could be functionally targeted (Dey et al., 2003; Delmore et al., 2011). This interaction is mediated by P-TEFb, which facilitates transcriptional elongation and Pol II promoter pause release (Bisgrove et al., 2007; Rahl et al., 2010). To target MYC function, JQ1 a small molecule inhibitor of BET bromodomains was evaluated for therapy. In multiple myeloma, which is characterized by chromosomal aberrations including MYC amplification, JQ1 showed significant effects in abrogating MYC oncogenic function and also reduced MYC expression (Delmore et al., 2011). JQ1 has also been shown to have anti-proliferative effects in acute lymphoblastic leukemia (Ott et al., 2012). While, JQ1 has shown promise in targeting MYC, other inhibitors of MYC downstream targets may also be of therapeutic interest.

**FIGURE 4:**
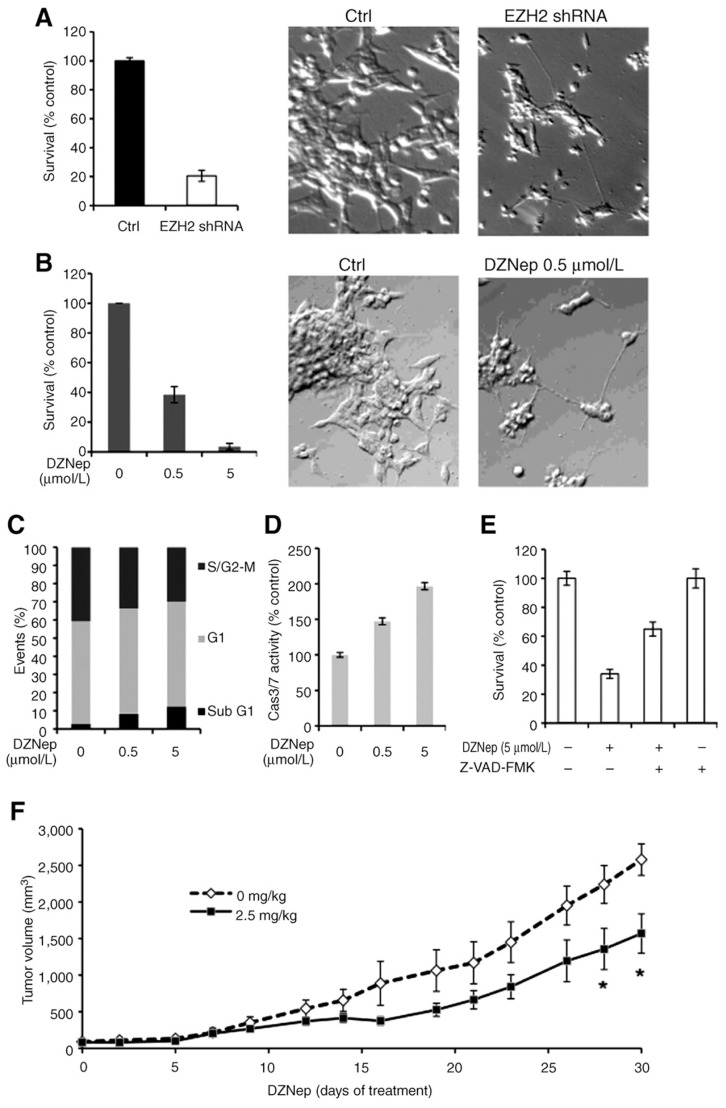
**Decrease of *EZH2* affects NB cell growth and induces the neurites (Wang et al., 2012)**. **(A)** Cell survival in KCNR cells after a 3-day infection with *EZH2* or non-target shRNA lentivirus was assessed using a Cell-Titer Blue assay (left). The percentage of surviving cells was normalized by the absorbance value of the non-target shRNA-infected cells (control). Representative images (×200) of the non-target shRNA-infected cells (ctrl, middle), EZH2 shRNA–infected cells (EZH2 shRNA, right). **(B)** KCNR cells were treated with different concentration of DZNep for 96 h. MTS assay was used to detected cell survival (left). The percentage of surviving cells was normalized by the absorbance value of the non-treated cells. Representative images (×200) of the non-treated cells (ctrl, middle) and 0.5 μmol/L DZNep-treated cells (DZNep 0.5 μmol/L, right). **(C)** KCNR cells were treated with different concentration of DZNep for 96 h. The cells were stained with propidium iodide and analyzed by flow cytometry. The data showed percentage of events in sub-G_1_, G_1_, and S/G_2_–M phase. **(D)** Caspase 3/7 activities were assessed after 48 h with different concentration of DZNep in KCNR cells. The percentage of caspase 3/7 activity was graphed after normalization to non-treated cells. **(E)** KCNR cells were treated with 5 μmol/L DZNep in the absence or presence of 100 μmol/L pan caspase inhibitor, Z-VAD-FMK, for 72 h. The percentage of surviving cells was graphed after normalization to untreated control. **(F)** Mice were treated with or without DZNep (2.5 mg/kg) twice a day, 3 days per week for 4 weeks. The mean tumor volumes are plotted using the SEM. The time points with significant differences (*P* < 0.05) are indicated with an asterisk. Adapted from Wang et al. (2012). © 2012 American Association for Cancer Research.

As MYC and MYCN have been shown to induce EZH2 expression and activity (Zhang et al., 2005; Neri et al., 2012) it is reasonable to assume that this also occurs in NB tumors. Targeting EZH2 may be another avenue to more robustly de-repress a subset of genes suppressed due to MYCN stimulation of EZH2. In NB cells inhibition of MYCN expression by targeted small interfering RNAs (siRNAs) leads to decreases in EZH2 protein levels which is accompanied by a decrease in the global levels of H3K27me3 (**Figure 4**; Wang et al., 2012). The importance of targeting EZH2 in NB was first noted with our finding that targeted inhibition of EZH2 leads to induction of genes having tumor suppressive or differentiation inducing capacity (Wang et al., 2012). In this study, targeted inhibition of EZH2 using shRNA (**Figure 4A**) or a small molecule inhibitor of EZH2, 3-Deazaneplanocin A (DZNep; **Figure 4B**) led to decreased growth and induction of neurites. Mechanistically this was also accompanied by induction of cell death (**Figure 4C**) which was due to increased levels of activated caspase 3/7 activity (**Figure 4D**) since the apoptotic effects of DZNep could be reversed by a caspase 3/7 inhibitor (**Figure 4E**). The finding that DZNep inhibited the growth of NB tumor xenografts is promising (**Figure 4F**). However, all mice still succumbed to tumors suggesting that combination therapies may be required to more efficaciously block tumor growth.

MYC’s dysregulation of epigenetic writers, readers, or erasers thus provides a novel avenue that can be therapeutically developed since many of these proteins have targetable enzyme activities (Lawlor and Thiele, 2012).

## Conflict of Interest Statement

The authors declare that the research was conducted in the absence of any commercial or financial relationships that could be construed as a potential conflict of interest.

## References

[B1] Allard S. Utley R. T. Savard J. Clarke A. Grant P. Brandl C. J. (1999). NuA4, an essential transcription adaptor/histone H4 acetyltransferase complex containing Esa1p and the ATM-related cofactor Tra1p. *EMBO J.* 18 5108–5119 10487762 10.1093/emboj/18.18.5108 PMC1171581

[B2] Ayer D. E. (1999). Histone deacetylases: transcriptional repression with SINers and NuRDs. *Trends Cell Biol.* 9 193–198 10322454 10.1016/s0962-8924(99)01536-6

[B3] Bernstein B. E. Meissner A. Lander E. S. (2007). The mammalian epigenome. *Cell* 128 669–681 17320505 10.1016/j.cell.2007.01.033

[B4] Bintu L. Ishibashi T. Lander E. S. (2012). Nucleosomal elements that control the topography of the barrier to transcription. *Cell* 151 738–749 23141536 10.1016/j.cell.2012.10.009 PMC3508686

[B5] Bisgrove D. A. Mahmoudi T. Henklein P. Verdin E. (2007). Conserved P-TEFb-interacting domain of BRD4 inhibits HIV transcription. *Proc. Natl. Acad. Sci. U.S.A.* 104 13690–13695 17690245 10.1073/pnas.0705053104 PMC1959443

[B6] Calao M. Sekyere E. O. Cui H. J. Cheung B. B. Thomas W. D. Keating J. (2012).Direct effects of Bmi1 on p53 protein stability inactivates oncoprotein stress responses in embryonal cancer precursor cells at tumor initiation. *Oncogene* 10.1038/onc.2012.368 [Epub ahead of print] 22907436

[B7] Cetinkaya C. Hultquist A. Su Y. Wu S. Bahram F. Påhlman S. (2007). Combined IFN-gamma and retinoic acid treatment targets the N-Myc/Max/Mad1 network resulting in repression of N-Myc target genes in MYCN-amplified neuroblastoma cells. *Mol. Cancer Ther.* 6 2634–2641 17938259 10.1158/1535-7163.MCT-06-0492

[B8] Chan H. M La Thangue N. B. (2001). p300/CBP proteins: HATs for transcriptional bridges and scaffolds. *J. Cell Sci.* 114(Pt 13) 2363–2373 11559745 10.1242/jcs.114.13.2363

[B9] Cheng S. W. Davies K. P. Yung E. Beltran R. J. Yu J. Kalpana G. V. (1999). c-MYC interacts with INI1/hSNF5 and requires the SWI/SNF complex for transactivation function. *Nat. Genet.* 22 102–105 10319872 10.1038/8811

[B10] Cirillo L. A. Lin F. R. Cuesta I. Friedman D. Jarnik M. Zaret K. S. (2002). Opening of compacted chromatin by early developmental transcription factors HNF3 (FoxA) and GATA-4. *Mol. Cell* 9 279–289 11864602 10.1016/s1097-2765(02)00459-8

[B11] Cole M. D. Cowling V. H. (2008). Transcription-independent functions of MYC: regulation of translation and DNA replication. *Nat. Rev. Mol. Cell Biol.* 9 810–815 18698328 10.1038/nrm2467 PMC3880805

[B12] Cotterman R. Jin V. X. Krig S. R. Lemen J. M. Wey A Farnham P. J. (2008). N-Myc regulates a widespread euchromatic program in the human genome partially independent of its role as a classical transcription factor. *Cancer Res.* 68 9654–9662 19047142 10.1158/0008-5472.CAN-08-1961 PMC2637654

[B13] Cowling V. H. Cole M. D. (2010). Myc regulation of mRNA cap methylation. *Genes Cancer* 1 576–579 21170289 10.1177/1947601910378025 PMC3002257

[B14] Cui H. Hu B. Li T. Ma J. Alam G. Gunning W. T. (2007). Bmi-1 is essential for the tumorigenicity of neuroblastoma cells. *Am. J. Pathol.* 170 1370–1378 17392175 10.2353/ajpath.2007.060754 PMC1829469

[B15] Delmore J. E. Issa G. C. Lemieux M. E. Rahl P. B. Shi J. Jacobs H. M. (2011). BET bromodomain inhibition as a therapeutic strategy to target c-Myc. *Cell* 146 904–917 21889194 10.1016/j.cell.2011.08.017 PMC3187920

[B16] Dey A. Chitsaz F. Abbasi A. Misteli T. Ozato K. (2003). The double bromodomain protein Brd4 binds to acetylated chromatin during interphase and mitosis. *Proc. Natl. Acad. Sci. U.S.A.* 100 8758–8763 12840145 10.1073/pnas.1433065100 PMC166386

[B17] Dhalluin C.J. Carlson E. Zeng L. He C. Aggarwal A. K. Zhou M. M. (1999). Structure and ligand of a histone acetyltransferase bromodomain. *Nature* 399 491–496 10365964 10.1038/20974

[B18] Filion G. J. van Bemmel J. G. Braunschweig U. Talhout W. Kind J. Ward L. D. (2010). Systematic protein location mapping reveals five principal chromatin types in *Drosophila* cells. *Cell* 143 212–224 20888037 10.1016/j.cell.2010.09.009 PMC3119929

[B19] Frank S. R. Parisi T. Taubert S. Fernandez P. Fuchs M. Chan H. M. (2003). MYC recruits the TIP60 histone acetyltransferase complex to chromatin. *EMBO Rep.* 4 575–580 12776177 10.1038/sj.embor.embor861 PMC1319201

[B20] Goto S. Umehara S. Gerbing R. B. Stram D. O. Brodeur G. M. Seeger R. C. (2001). Histopathology (International Neuroblastoma Pathology Classification) and MYCN status in patients with peripheral neuroblastic tumors: a report from the Children’s Cancer Group. *Cancer* 92 2699–2708 11745206 10.1002/1097-0142(20011115)92:10<2699::aid-cncr1624>3.0.co;2-a

[B21] Guccione E. Martinato F. Finocchiaro G. Luzi L. Tizzoni L. Dall’ Olio V. (2006). Myc-binding-site recognition in the human genome is determined by chromatin context. *Nat. Cell Biol.* 8 764–770 16767079 10.1038/ncb1434

[B22] Iraci N. Diolaiti D. Papa A. Porro A. Valli E. Gherardi S. (2011). A SP1/MIZ1/MYCN repression complex recruits HDAC1 at the TRKA and p75NTR promoters and affects neuroblastoma malignancy by inhibiting the cell response to NGF. *Cancer Res.* 71 404–412 21123453 10.1158/0008-5472.CAN-10-2627

[B23] Kim J. Woo A. J. Chu J. Snow J. W. Fujiwara Y. Kim C. G. (2010). A Myc network accounts for similarities between embryonic stem and cancer cell transcription programs. *Cell* 143 313–324 20946988 10.1016/j.cell.2010.09.010 PMC3018841

[B24] Klempnauer K. H. (1989). Association of v-myc protein with chromatin. *Oncogene* 4 115–118 2644610

[B25] Knoepfler P. S. Eisenman R. N. (1999). Sin meets NuRD and other tails of repression. *Cell* 99 447–450 10589671 10.1016/s0092-8674(00)81531-7

[B26] Knoepfler P. S. Zhang X. Y. Cheng P. F. Gafken P. R. McMahon S. B. Eisenman R. N. (2006). Myc influences global chromatin structure. *EMBO J.* 25 2723–2734 16724113 10.1038/sj.emboj.7601152 PMC1500848

[B27] Laherty C. D. Yang W. M. Sun J. M. Davie J. R. Seto E. Eisenman R. N. (1997). Histone deacetylases associated with the mSin3 corepressor mediate mad transcriptional repression. *Cell* 89 349–356 9150134 10.1016/s0092-8674(00)80215-9

[B28] Lawlor E. R. Thiele C. J. (2012). Epigenetic changes in pediatric solid tumors: promising new targets. *Clin. Cancer Res.* 18 2768–2779 22589485 10.1158/1078-0432.CCR-11-1921 PMC3691809

[B29] Lehmann L. Ferrari R. Vashisht A. A. Wohlschlegel J. A. Kurdistani S. K. Carey M. (2012). Polycomb repressive complex 1 (PRC1) disassembles RNA polymerase II preinitiation complexes. *J. Biol. Chem.* 287 35784–35794 22910904 10.1074/jbc.M112.397430 PMC3476247

[B30] Lin C. Y. Loven J. Rahl P. B. Paranal R. M. Burge C. B. Bradner J. E. (2012). Transcriptional amplification in tumor cells with elevated c-Myc. *Cell* 151 56–67 23021215 10.1016/j.cell.2012.08.026 PMC3462372

[B31] Lucarelli E. Kaplan D. R. Thiele C. J. (1995). Selective regulation of TrkA and TrkB receptors by retinoic acid and interferon-gamma in human neuroblastoma cell lines. *J. Biol. Chem.* 270 24725–24731 7559588 10.1074/jbc.270.42.24725

[B32] Malynn B. A. de Alboran I. M. O’Hagan R. C. Bronson R. Davidson L. DePinho R. A. (2000). N-myc can functionally replace c-myc in murine development, cellular growth, and differentiation. *Genes Dev.* 14 1390–1399 10837031 PMC316670

[B33] Martinez-Cerdeno V. Lemen J. M. O’Hagan R. C. Bronson R. Davidson L. DePinho R. A. (2012). N-Myc and GCN5 regulate significantly overlapping transcriptional programs in neural stem cells. *PLoS ONE* 7:e39456 10.1371/journal.pone.0039456 PMC3383708 22745758

[B34] McEwan I. J. Dahlman-Wright K. Ford J. Wright A. P (1996). Functional interaction of the c-Myc transactivation domain with the TATA binding protein: evidence for an induced fit model of transactivation domain folding. *Biochemistry* 35 9584–9593 8755740 10.1021/bi960793v

[B35] McMahon S. B. Van Buskirk H. A. Dugan K. A. Copeland T. D. Cole M. D. (1998). The novel ATM-related protein TRRAP is an essential cofactor for the c-Myc and E2F oncoproteins. *Cell* 94 363–374 9708738 10.1016/s0092-8674(00)81479-8

[B36] McMahon S. B. Wood M. A. Cole M. D. (2000). The essential cofactor TRRAP recruits the histone acetyltransferase hGCN5 to c-Myc. *Mol. Cell. Biol.* 20 556–562 10611234 10.1128/mcb.20.2.556-562.2000 PMC85131

[B37] Neri F. Zippo A. Krepelova A. Cherubini A. Rocchigiani M. Oliviero S. (2012).Myc regulates the transcription of the PRC2 gene to control the expression of developmental genes in embryonic stem cells. *Mol. Cell. Biol.* 32 840–851 22184065 10.1128/MCB.06148-11 PMC3272981

[B38] Nie Z. Hu G. Wei G. Cui K. Yamane A. Resch W. (2012). c-Myc is a universal amplifier of expressed genes in lymphocytes and embryonic stem cells. *Cell* 151 68–79 23021216 10.1016/j.cell.2012.08.033 PMC3471363

[B39] Ochiai H. Takenobu H. Nakagawa A. Yamaguchi Y. Kimura M. Ohira M. (2010). Bmi1 is a MYCN target gene that regulates tumorigenesis through repression of KIF1Bbeta and TSLC1 in neuroblastoma. *Oncogene* 29 2681–2690 20190806 10.1038/onc.2010.22

[B40] Ogryzko V. V. Schiltz R. L. Russanova V. Howard B. H. Nakatani Y. (1996). The transcriptional coactivators p300 and CBP are histone acetyltransferases. *Cell* 87 953–959 8945521 10.1016/s0092-8674(00)82001-2

[B41] Ott C. J. Kopp N. Bird L. Paranal R. M. Qi J. Bowman T. (2012). BET bromodomain inhibition targets both c-Myc and IL7R in high-risk acute lymphoblastic leukemia. *Blood* 120 2843–2852 22904298 10.1182/blood-2012-02-413021 PMC3466965

[B42] Park J. R. Villablanca J. G. London W. B. Gerbing R. B. Haas-Kogan D. Adkins E. S. (2009). Outcome of high-risk stage 3 neuroblastoma with myeloablative therapy and 13-cis-retinoic acid: a report from the Children’s Oncology Group. *Pediatr. Blood Cancer* 52 44–50 18937318 10.1002/pbc.21784 PMC2731719

[B43] Patel J. H. Du Y. Ard P. G. Phillips C. Carella B. Chen C. J. (2004). The c-MYC oncoprotein is a substrate of the acetyltransferases hGCN5/PCAF and TIP60. *Mol. Cell. Biol.* 24 10826–10834 15572685 10.1128/MCB.24.24.10826-10834.2004 PMC533976

[B44] Rahl P. B. Lin C. Y. Seila A. C. Flynn R. A. McCuine S. Burge C. B. (2010). c-Myc regulates transcriptional pause release. *Cell* 141 432–445 20434984 10.1016/j.cell.2010.03.030 PMC2864022

[B45] Romero O. A. Setien F. John S. Gimenez-Xavier P. Gómez-López G. Pisano D. (2012). The tumour suppressor and chromatin-remodelling factor BRG1 antagonizes Myc activity and promotes cell differentiation in human cancer. *EMBO Mol. Med.* 4 603–616 22407764 10.1002/emmm.201200236 PMC3407948

[B46] Schwab M. Alitalo K. Klempnauer K. H. Varmus H. E. Bishop J. M. Gilbert F. (1983). Amplified DNA with limited homology to myc cellular oncogene is shared by human neuroblastoma cell lines and a neuroblastoma tumour. *Nature* 305 245–248 6888561 10.1038/305245a0

[B47] Simon J. A. Kingston R. E. (2009). Mechanisms of polycomb gene silencing: knowns and unknowns. *Nat. Rev. Mol. Cell Biol.* 10 697–708 19738629 10.1038/nrm2763

[B48] Soufi A. Donahue G. Zaret K. S. (2012). Facilitators and impediments of the pluripotency reprogramming factors’ initial engagement with the genome. *Cell* 151 994–1004 23159369 10.1016/j.cell.2012.09.045 PMC3508134

[B49] Stone J. de Lange T. Ramsay G. Jakobovits E. Bishop J. M. Varmus H. (1987). Definition of regions in human c-myc that are involved in transformation and nuclear localization. *Mol. Cell. Biol.* 7 1697–1709 3299053 10.1128/mcb.7.5.1697-1709.1987 PMC365270

[B50] Strahl B. D. Allis C. D. (2000). The language of covalent histone modifications. *Nature* 403 41–45 10638745 10.1038/47412

[B51] Telford D. J. Stewart B. W. (1990). Structural features of the amplified N-myc oncogene detected by micrococcal nuclease digestion of neuroblastoma cell nuclei. *Biochem.Int.* 21 1025–1032 2080918

[B52] Thiele C. J. Reynolds C. P. Israel M. A. (1985). Decreased expression of N-myc precedes retinoic acid-induced morphological differentiation of human neuroblastoma. *Nature* 313 404–406 3855502 10.1038/313404a0

[B53] Valentijn L. J. Koster J. Haneveld F. Aissa R. A. van Sluis P. Broekmans M. E. (2012). Functional MYCN signature predicts outcome of neuroblastoma irrespective of MYCN amplification. *Proc. Natl. Acad. Sci. U.S.A.* 109 19190–19195 23091029 10.1073/pnas.1208215109 PMC3511149

[B54] Vervoorts J. Luscher-Firzlaff J. M. Rottmann S. Lilischkis R. Walsemann G. Dohmann K. (2003). Stimulation of c-MYC transcriptional activity and acetylation by recruitment of the cofactor CBP. *EMBO Rep.* 4 484–490 12776737 10.1038/sj.embor.embor821 PMC1319176

[B55] Wang C. Liu Z. Woo C. W. Li Z. Wang L. Wei J. S. (2012). EZH2 Mediates epigenetic silencing of neuroblastoma suppressor genes CASZ1, CLU, RUNX3, and NGFR. *Cancer Res.* 72 315–324 22068036 10.1158/0008-5472.CAN-11-0961 PMC3487161

[B56] Wood M. A. McMahon S. B. Cole M. D. (2000).An ATPase/helicase complex is an essential cofactor for oncogenic transformation by c-Myc. *Mol. Cell* 5 321–330 10882073 10.1016/s1097-2765(00)80427-x

[B57] Wu C. H. van Riggelen J. Yetil A. Fan A. C. Bachireddy P. Felsher D. W. (2007). Cellular senescence is an important mechanism of tumor regression upon c-Myc inactivation. *Proc. Natl. Acad. Sci. U.S.A.* 104 13028–13033 17664422 10.1073/pnas.0701953104 PMC1941831

[B58] Zaret K. S. Carroll J. S. (2011). Pioneer transcription factors: establishing competence for gene expression. *Genes Dev.* 25 2227–2241 22056668 10.1101/gad.176826.111 PMC3219227

[B59] Zhang X. Y. DeSalle L. M. Patel J. H. Capobianco A. J. Yu D. Thomas-Tikhonenko A. (2005). Metastasis-associated protein 1 (MTA1) is an essential downstream effector of the c-MYC oncoprotein. *Proc. Natl. Acad. Sci. U. S. A.* 102 13968–13973 16172399 10.1073/pnas.0502330102 PMC1236531

